# Performance Evaluation of the VITEK2 and Sensititre Systems to Determine Colistin Resistance and MIC for *Acinetobacter baumannii*

**DOI:** 10.3390/diagnostics12061487

**Published:** 2022-06-17

**Authors:** Hae-Sun Chung, Soo-Kyung Kim, Chorong Hahm, Miae Lee

**Affiliations:** 1 Department of Laboratory Medicine, Ewha Womans University College of Medicine, Seoul 07985, Korea; sunny0521.chung@ewha.ac.kr (H.-S.C.); skkim1@ewha.ac.kr (S.-K.K.); crhahm@eonelab.co.kr (C.H.); 2 Ewha Education and Research Center for Infection, Seoul 07985, Korea; 3 Department of Laboratory Medicine, Eone Laboratories, Incheon 22014, Korea

**Keywords:** *Acinetobacter baumannii*, colistin, antimicrobial susceptibility test

## Abstract

Performances of the colistin antimicrobial susceptibility testing (AST) systems of *Acinetobacter baumannii* vary depending on the manufacturer, and data on colistin-resistant *A. baumannii* are limited. We evaluated the VITEK2 and Sensititre systems to determine colistin resistance and minimum inhibitory concentration (MIC) for *A. baumannii* isolated from a clinical microbiology laboratory. A total of 213 clinical *A. baumannii* isolates were tested, including 81 colistin-resistant *A. baumannii*. ASTs were performed using the VITEK2 and Sensititre systems according to the manufacturer’s instructions. Reference MICs for colistin were determined using the manual broth microdilution method (BMD). The results of the two AST methods were compared with the BMD results. VITEK2 and Sensititre systems showed category agreements of 95.3% and 99.1%, respectively. VITEK2 had a relatively high very major error (VME) rate (9.9%). Sensititre reported higher MICs than the reference method for the susceptible isolates and showed low essential agreement. In conclusion, the automated systems investigated in this study showed good category agreements for colistin AST of *A. baumannii*. However, VITEK2 had a high VME rate, and Sensititre had differences in MIC results. Colistin AST remains a challenging task in the clinical laboratory.

## 1. Introduction

Colistin is a last-line therapeutic option for infections caused by multidrug-resistant Gram-negative bacteria, including *Acinetobacter baumannii*. However, colistin-resistant strains of Gram-negative bacteria have been reported globally [1,2]. According to the national antimicrobial resistance surveillance data in Korea [3], the colistin resistance rate in *A. baumannii* is almost 0%. In 2020, it suddenly increased to 7.2%, which was attributed to an outbreak at a sentinel hospital [4]. However, a systematic review and meta-analysis reported an overall resistance rate of 7.8% (95% confidential interval 4.0–14.7%) [5].

Considering the increasing colistin resistance and toxicity, accurate antimicrobial susceptibility testing (AST) of colistin is particularly important for the successful treatment of multidrug-resistant *A. baumannii* infections. However, AST of colistin is challenging for clinical laboratories [6,7,8]. A joint recommendation by the Clinical and Laboratory Standards Institute (CLSI) and European Committee on Antimicrobial Susceptibility Testing (EUCAST) released in 2016 recommended the ISO-20776 standard broth microdilution (BMD) method for colistin minimum inhibitory concentration (MIC) testing [9]. Notably, there are significant differences in the results between methods other than the standard method. Despite the recommendation, in practice, it is extremely difficult to follow the standard method in clinical laboratories because it is laborious, and manual preparation may lead to significant errors. Therefore, in clinical laboratories, AST is performed by using commercially available automated systems or gradient strips [10]. Although most of the automated systems follow the standard method, their performances vary depending on the manufacturer. Furthermore, automated systems commonly used in clinical laboratories have not been sufficiently verified for the AST of colistin; hence, there are limited data on colistin-resistant *A. baumannii*.

In this study, we evaluated the VITEK2 system (bioMérieux, Marcy-l’Étoile, France) and Sensititre system (Thermo Scientific, Waltham, MA, USA) to determine colistin resistance and MIC for *A. baumannii* isolated from a clinical microbiology laboratory.

## 2. Materials and Methods

*A. baumannii* isolates were collected from a clinical microbiology laboratory of a tertiary university hospital from April 2015 until October 2020. In total, 213 clinical *A. baumannii* isolates were tested. To include as many colistin-resistant *A. baumannii* isolates as possible, 75 *A. baumannii* isolates identified as colistin-resistant by the VITEK2 system were included. Bacterial identification was performed using VITEK2 system or VITEK MS (bioMérieux, Marcy-l’Étoile, France).

AST was performed using the VITEK2 and Sensititre systems using an N225 card and a DKMGN plate, respectively, according to the manufacturer’s instructions. The MIC ranges for colistin on the N225 card and DKMGN plate were ≤0.5 μg/mL to ≥16 μg/mL and ≤0.25 μg/mL to >8 μg/mL in doubling dilutions, respectively. Reference MICs for colistin were determined using manual BMD according to CLSI guidelines. The BMD MIC test range was 0.25 μg/mL to 256 μg/mL. Susceptibility results for colistin were interpreted according to the CLSI guidelines as follows: ≤2 μg/mL indicated susceptibility, and ≥4 μg/mL indicated resistance [11]. For BMD and Sensititre quality control, *Escherichia coli* ATCC 25922 (MIC 0.25–2 μg/mL) and *Pseudomonas aeruginosa* ATCC 27853 (MIC 0.5–4 μg/mL) were tested according to CLSI guidelines and manufacturer’s instructions [11]. For VITEK2 quality control, *P. aeruginosa* ATCC 27853 (MIC 0.5–2 μg/mL) was tested according to manufacturer’s instructions. All control MICs were within acceptable ranges.

The results of the two AST methods were compared with those obtained using BMD as the reference method. The sensitivity, specificity, and positive and negative predictive values (PPV and NPV, respectively) of each method for the detection of colistin resistance in *A. baumannii* were calculated to evaluate the performance. Agreement was assessed based on a comparison of category (qualitative) and MIC (quantitative). Category agreement was defined as a case in which the results of the interpretive category (susceptible, intermediate, or resistant) were in agreement. A major error (ME) occurred when an isolate was categorized as resistant by the test method but susceptible by the reference method (false-resistant result). The ME rates were calculated using the number of isolates that were susceptible (according to BMD) as the denominator. A very major error (VME) occurred when an isolate was categorized as susceptible by the testing method but resistant by the reference method (false-susceptible result). The VME rates were calculated using the number of isolates resistant (according to BMD) as the denominator. Essential agreement was defined as MIC results within a two-fold dilution (±1 doubling dilution). It was analyzed by excluding values outside the determinable range due to uncertainty and by including the lowest or highest values outside the determinable range, assuming that they were in agreement. Agreements were analyzed between each testing method and compared with the reference method. To allow comparison of essential agreement between VITEK2 and Sensititre, VITEK2 MICs of ≤0.5 μg/mL and ≥16 μg/mL were considered as 0.5 and 16 μg/mL, and Sensititre MICs of >8 μg/mL were considered as 16 μg/mL, respectively. Acceptable agreement for the testing methods compared with BMD was defined as essential agreement ≥90%, category agreement ≥90%, VME ≤ 1.5%, and ME ≤ 3%, as described by the CLSI [12]. This study was approved by the Institutional Review Board of the Ewha Womans University Mokdong Hospital, which waived the requirement for informed consent (IRB File No. EUMC 2019-09-013-003).

## 3. Results

The colistin MIC determined via BMD ranged from 0.5 to >256 μg/mL, and 81 isolates were resistant to colistin. Thirty-two isolates had MIC values exceeding 256 μg/mL.

The testing methods were compared with BMD as a reference method (Table 1). Both testing methods showed category agreements above 95%, satisfying the acceptance criteria (≥90%). VITEK2 had a relatively high VME rate (9.9%), which was beyond the acceptable range (≤1.5%). Sensititre showed that all categories matched except for one VME and one ME.

When essential agreements were analyzed, excluding values outside the determinable range, only a few isolates remained in the denominator to calculate the essential agreements, namely 17 and 146 for VITEK2 and Sensititre, respectively, and the essential agreements were 29.4% (5/17) and 42.5% (62/146) for VITEK2 and Sensititre, respectively. When essential agreements were calculated by assuming that the highest and lowest MIC concentrations were in agreement, the essential agreements were 90.1% (192/213) and 60.6% (129/213) for VITEK2 and Sensititre, respectively. The low essential agreement of Sensititre was prominent in the susceptible group, which was 47.0% (62/132).

The distributions of MICs determined via BMD and the two testing methods are shown in Table 2. Sixty-eight isolates with MIC ≥ 16 μg/mL according to VITEK2 all had MICs ≥ 16 μg/mL (range: 16–>256 μg/mL) by BMD except for two isolates. Sixty-seven isolates with MIC > 8 μg/mL according to Sensititre all had MICs ≥ 8 μg/mL (range: 8–>256 μg/mL) when tested using BMD. Sensititre reported higher MICs for isolates with MICs ≤ 2 μg/mL than BMD reported: +1 dilution; 58 isolates, +2 dilution; 69 isolates, and +3; and 1 isolate.

The performance of the testing methods for detecting colistin resistance in *A. baumannii* is shown in Table 3. Both testing methods showed a high sensitivity, specificity, PPV, and NPV of >90%. For Sensititre, all values were approximately 99%. The sensitivity of VITEK2 was relatively low (90.1%).

Table 4 shows the comparison results of VITEK2 and Sensititre; their category agreement was high at 94.4%, but the essential agreement was low at 54.0%. The MICs determined via Sensititre were higher than those determined via VITEK2; only 5.6% (12 isolates) of Sensititre results showed lower MICs than the VITEK2 results. Of note, there were 12 discordant results (Table 5).

## 4. Discussion

In this study, two automated systems were evaluated, and differences in performance were observed. Except for essential agreement, which was difficult to evaluate accurately, Sensititre showed good performance, satisfying the AST performance requirements. However, the performance of VITEK2 was not appropriate for use in clinical laboratories because the VME was beyond the acceptable range.

When colistin resistance is extremely rare, it is rare to obtain false susceptible results (VME). However, as colistin resistance increases, the risk of VME also increases. VME is considered the most crucial error in AST because ineffective drugs may be administered to the patient, leading to treatment failure. It is notable that misinterpretation of colistin AST, especially by gradient strips or automated AST systems, may lead to treatment failure and even mortality [13,14]. Therefore, the high VME rate of VITEK2 is an important issue that must be addressed.

Sensititre reported higher MICs than the reference method for the susceptible isolates. This may not be a clinically significant problem because the results of the interpretive category were consistent with those of the reference method. However, differences in MIC results are clearly a problem with the testing method, and higher MIC results may also increase the risk of ME. Even if the 67 isolates with MICs > 8 μg/mL according to Sensititre and MICs ≥ 8 μg/mL (range: 8–>256 μg/mL) according to BMD were in agreement, the essential agreement of Sensititre did not exceed 90%. This does not satisfy AST performance requirements.

Both testing methods showed good sensitivity, specificity, PPV, and NPV for detecting colistin resistance in *A. baumannii*. However, the sensitivity of VITEK2 is relatively low; therefore, colistin-resistant *A. baumannii* may be overlooked by VITEK2, resulting in VMEs in the VITEK2 system.

In previous studies, the performance of colistin AST for *A. baumannii* in automated systems has been inconsistent. VITEK2 has been previously reported to be a reliable testing method for colistin in *A. baumannii* [15,16]. In Dafopoulou et al.’s study, 18 colistin-resistant *A. baumannii* isolates were evaluated, none of which showed VME [16]. However, more recent studies have shown that VITEK2 has an unreliable performance [17,18,19]. A high VME rate is a prominent problem: three studies reported VME rates of 22.2% (6/27) by Girardello et al., 37.9% (11/29) by Vourli et al., and 53.1% (17/32) by Khurana et al. These study results show higher VME rate of VITEK2 system than our study (VME rate 9.9%). Lower VME rate of this study compared to recent studies might be because this study included more colistin-resistant isolates. In this study, we included as many colistin-resistant *A. baumannii* isolates as possible, and 81 colistin-resistant isolates were tested, whereas previous studies included colistin-resistant isolates ranging from 8–32 isolates [16,17,18,19,20,21]. In previous studies, the essential agreement and category agreement were also low, ranging from 71–89% and 86–93%, respectively [17,18,19]. The essential agreement and category agreement of VITEK2 in this study were 90.1% and 95.3%, respectively, which were slightly higher than in previous studies.

Katip et al. discussed that *Klebsiella pneumoniae* and *E. coli* have a very good correlation between BMD and VITEK2 in very low (≤0.5 μg/mL) or very high (≥16 μg/mL) MICs and BMD might be unnecessary for these pathogens within the MIC ranges [10]. However, we identified VME in *A. baumannii* with very low VITEK2 MIC (≤0.5 μg/mL). Girardello et al. claimed that all colistin MICs should be confirmed for *A. baumannii,* and our study results are consistent with this opinion [17].

The essential agreement, category agreement, and VME rate of Sensititre compared to BMD were 90.1%, 99.1%, and 1.2%, respectively. Sensititre, as an automated system, was recently introduced and is yet to be used in clinical laboratories. Therefore, there were only a limited number of studies performed on Sensititre with colistin-resistant *A. baumannii* isolates. Previous studies also reported a high rate of essential agreement and category agreement of Sensititre with the reference BMD of 91% and 91–100%, respectively [20,21]. Previous studies reported a 0% VME rate, which was similar to our study [20,21].

In this study, BMD recorded MICs up to 256 μg/mL, which is a significantly higher concentration than that reported in other studies. As a result, 27 isolates with MICs exceeding 256 μg/mL were detected. In clinical laboratories, AST is usually performed with commercially available automated systems, and the measurement range is usually up to 16 μg/mL. In this study, VITEK2 and Sensititre were up to 16 μg/mL and 8 μg/mL, respectively. Therefore, it is difficult to recognize high-level resistance. Although the mechanisms underlying colistin resistance in *A. baumannii* are complex and not completely understood, it can be expected that the level of resistance will differ depending on the resistance mechanism. For example, mutations in the *lpxA*, *lpxC*, and *lpxD* genes of *A. baumannii* lead to inactivation of lipid A biosynthesis; thus, a complete loss of lipopolysaccharide occurs with subsequent loss of the polymyxin target, resulting in extremely high colistin MICs [1]. Therefore, the diagnosis of high-level colistin resistance may be necessary to identify the mechanism of resistance.

There were some limitations to the selection of the test isolates. In this study, *A. baumannii* isolates detected in the clinical laboratory were studied, but since we tried to include as many colistin-resistant *A. baumannii* isolates as possible, there is a difference from the prevalence in actual clinical settings. Excluding duplicate isolates, the colistin resistance rate of *A. baumannii* in this hospital between 2014 and 2018 was 1.9% (84/4467) [22].

In 2020, the CLSI guidelines changed the interpretive criteria; reporting of isolates with MICs ≤ 2 μg/mL was changed from susceptible to intermediate [23]. If the updated guideline is applied, the agreement does not change, and all VMEs and MEs become minor errors. To analyze VME and ME, which have greater clinical significance than minor errors, in this study, the 2019 guideline was applied but not the updated guidelines.

In conclusion, the automated systems investigated in this study showed good category agreements for colistin AST of *A. baumannii*. However, VITEK2 had a high VME rate, and Sensititre had differences in MIC results.

Colistin AST remains a challenging task in the laboratory. Caution should be taken when interpreting susceptibility results for colistin in *A. baumannii* using an automated system.

## Figures and Tables

**Table 1:** Agreements of colistin susceptibility results between different antimicrobial susceptibility testing methods and broth microdilution.

Testing Method	Category Agreement (%)	No. of Error	
		Very Major (Rate, %)	Major (Rate, %)
VITEK2	95.3 (203/213)	8 (9.9)	2 (1.5)
Sensititre	99.1 (211/213)	1 (1.2)	1 (0.8)

**Table 2:** Colistin MICs of VITEK2 and Sensititre system compared to broth microdilution.

Testing Method	MIC (μg/mL)	No. of Isolates with Colistin MIC (μg/mL) Determined by Broth Microdilution											Sum
		0.5	1	2	4	8	16	32	64	128	256	>256	
VITEK2	≤0.5	109	12	2				1		1		3	128
	1	1	3			1							5
	2	2		1							1	1	5
	4									1	2		3
	8									3		1	4
	≥16	2					2	3	4	17	13	27	68
Sensititre	0.5	1											1
	1	44											44
	2	69	14	3							1		87
	4		1					1	1	3	3		9
	8							1	1	3			5
	>8					1	2	2	2	16	12	32	67
	Sum	114	15	3	0	1	2	4	4	22	16	32	213

**Table 3:** The performance for detection of colistin resistance in *Acinetobacter baumannii* of different antimicrobial susceptibility testing methods.

Testing Method		BMD (No. of Isolates)		Sensitivity (%)	Specificity (%)	PPV (%)	NPV (%)
		S	R				
VITEK2	S	130	8	90.1	98.5	97.3	94.2
	R	2	73				
Sensititre	S	131	1	98.8	99.2	98.8	99.2
	R	1	80				

Abbreviations: BMD, broth microdilution; NPV, negative predictive value; PPV, positive predictive value; R, resistant; S, susceptible.

**Table 4:** Comparison of colistin MICs and susceptibility determined by the VITEK2 and Sensititre system.

		MIC (μg/mL)	Sensititre						Essential Agreement (%)	Category Agreement (%)
			S			R				
			0.5	1	2	4	8	>8		
VITEK2	S	≤0.5	1	41	80	1		5	54.0 (115/213)	94.4 (201/213)
		1			4			1		
		2		1	2	1		1		
	R	4				1		2		
		8				1	1	2		
		≥16		2	1	5	4	56		
		Sum	1	44	87	9	5	67		

Abbreviations: R, resistant; S, susceptible.

**Table 5:** Discordant results of the VITEK2 and Sensititre system.

No. of Isolates	MIC (Susceptibility)						Interpretation	
	VITEK2		Sensititre		BMD		VITEK2	Sensititre
1	≤0.5	(S)	4	(R)	1	(S)	True S	False R (Major error)
1	≤0.5	(S)	>8	(R)	32	(R)	False S (Very major error)	True R
1	≤0.5	(S)	>8	(R)	128	(R)	False S (Very major error)	True R
3	≤0.5	(S)	>8	(R)	>256	(R)	False S (Very major error)	True R
1	1	(S)	>8	(R)	8	(R)	False S (Very major error)	True R
1	2	(S)	4	(R)	256	(R)	False S (Very major error)	True R
1	2	(S)	>8	(R)	>256	(R)	False S (Very major error)	True R
2	≥16	(R)	1	(S)	0.5	(S)	False R (Major error)	True S
1	≥16	(R)	2	(S)	256	(R)	True R	False S (Very major error)

Abbreviations: BMD, broth microdilution; R, resistant; S, susceptible.

## Data Availability

Data of this study can be available on request.
